# Atrial Fibrillation Prediction from Critically Ill Sepsis Patients

**DOI:** 10.3390/bios11080269

**Published:** 2021-08-09

**Authors:** Syed Khairul Bashar, Eric Y. Ding, Allan J. Walkey, David D. McManus, Ki H. Chon

**Affiliations:** 1 Biomedical Engineering Department, University of Connecticut, Storrs, CT 06269, USA; syed.bashar@uconn.edu; 2 Division of Cardiology, University of Massachusetts Medical School, Worcester, MA 01655, USA; eric.ding@umassmed.edu (E.Y.D.); david.mcmanus@umassmed.edu (D.D.M.); 3 Department of Medicine, Boston University School of Medicine, Boston, MA 02118, USA; alwalkey@bu.edu

**Keywords:** sepsis, atrial fibrillation, prediction, heart rate variability, feature extraction, random forest, annotations

## Abstract

Sepsis is defined by life-threatening organ dysfunction during infection and is the leading cause of death in hospitals. During sepsis, there is a high risk that new onset of atrial fibrillation (AF) can occur, which is associated with significant morbidity and mortality. Consequently, early prediction of AF during sepsis would allow testing of interventions in the intensive care unit (ICU) to prevent AF and its severe complications. In this paper, we present a novel automated AF prediction algorithm for critically ill sepsis patients using electrocardiogram (ECG) signals. From the heart rate signal collected from 5-min ECG, feature extraction is performed using the traditional time, frequency, and nonlinear domain methods. Moreover, variable frequency complex demodulation and tunable Q-factor wavelet-transform-based time–frequency methods are applied to extract novel features from the heart rate signal. Using a selected feature subset, several machine learning classifiers, including support vector machine (SVM) and random forest (RF), were trained using only the 2001 Computers in Cardiology data set. For testing the proposed method, 50 critically ill ICU subjects from the Medical Information Mart for Intensive Care (MIMIC) III database were used in this study. Using distinct and independent testing data from MIMIC III, the SVM achieved 80% sensitivity, 100% specificity, 90% accuracy, 100% positive predictive value, and 83.33% negative predictive value for predicting AF immediately prior to the onset of AF, while the RF achieved 88% AF prediction accuracy. When we analyzed how much in advance we can predict AF events in critically ill sepsis patients, the algorithm achieved 80% accuracy for predicting AF events 10 min early. Our algorithm outperformed a state-of-the-art method for predicting AF in ICU patients, further demonstrating the efficacy of our proposed method. The annotations of patients’ AF transition information will be made publicly available for other investigators. Our algorithm to predict AF onset is applicable for any ECG modality including patch electrodes and wearables, including Holter, loop recorder, and implantable devices.

## 1. Introduction

Sepsis is a life-threatening, dysregulated response to infection and is the leading cause of death in the hospitals of the United States. Sepsis affects more than 1.5 million Americans yearly at an annual cost of over $20 billion [1]. Atrial fibrillation (AF) is a common and deadly complication of sepsis; it is associated with poor outcomes during hospitalization and confers risk for significant adverse events long thereafter [2]. The mechanisms of AF during sepsis are unclear and may involve rapid remodeling from infection as well as triggers from autonomic nervous system activation, fluid shifts, and electrolyte disturbances [3]. Patients with sepsis have sixfold higher risk of new-onset AF as compared with hospitalized patients without sepsis and similar cardiovascular risk factors. New-onset AF during sepsis is a common and deadly dysrhythmia during sepsis, affecting nearly 1 in 5 septic patients [4,5] and is associated with significant morbidity and mortality [6]. As a result, early prediction of AF during sepsis could potentially lead to AF intervention strategies, thereby minimizing poor hospital outcomes during sepsis.

For the past two decades, there have been many studies of AF prediction using electrocardiogram (ECG) signals outside of the ICU setting. In [7], the frequent occurrence of atrial premature beats prior to the onset of premature atrial contraction (PAC) was reported to be predictive. PAC is characterized by analyzing the quantities of atrial and ventricular ectopic beats from the RR intervals; an increase in atrial ectopic beats is reported in subjects prior to AF episodes [8]. In [9], correlation coefficients, time domain, frequency domain, power spectral densities, and P waves were used to predict paroxysmal AF (PAF). Spectral, bispectral, and nonlinear measurements from 30-min heart rate variability data were used in [10] to predict PAF events. Time domain, frequency domain, nonlinear, and bispectrum features were calculated from 15-min heart rate data; genetic-algorithm-based optimization and a support vector machine classifier were used to predict PAF in [11]. In [12], time, frequency, and nonlinear domain heart rate variability (HRV) features were extracted first, which were then fed into an SVM classifier; feature subset and classifier tuning were performed using nondominated sorting genetic algorithm III. A predictor based on the number of premature atrial complexes not followed by a regular RR interval, runs of atrial bigeminy and trigeminy, and the length of any short run of paroxysmal atrial tachycardia was presented in [13]. In [14], short-term heart rate variability-based features were extracted first; then, genetic-algorithm-based feature selection and *k*-nearest neighbor classifier were applied to predict PAF. An AF prediction algorithm based on nonlinear features calculated from the return map and difference map of HRV signals was reported in [15]. A symbolic dynamic approach known as footprint analysis was presented in [16] to investigate heart rate dynamics before PAF episodes. In [17], a combination of linear, time–frequency, and nonlinear analysis were performed on heart rate variability and a mixture of experts classification was used for PAF prediction.

However, the common factor for most of the above-mentioned methods is that they were developed and validated using the 2001 Computing in Cardiology (CinC) Challenge data set, as this is the only publicly available data set so far for AF prediction. Thus, the AF prediction studies are limited by the available data sets. In the CinC data set, PAF prediction is performed within the PAF subjects using the two ECG records (pre-AF and distant from AF data segments) from the same subject. Moreover, none of these methods examined AF prediction in critically ill ICU patients. The mechanisms of AF during sepsis may differ from other clinical scenarios; therefore, AF prediction algorithms may differ during sepsis [3,5]. As a result, the above-mentioned methods lack a prospective head-to-head evaluation with clinically derived real life data [17].

In order to address the novel challenges of AF prediction during sepsis, in this study, we present a machine learning approach for AF prediction for ICU patients with sepsis. We used traditional HRV parameters as well as novel time–frequency-based features to identify pre-AF ECG recordings from critically ill sepsis patients.

The major contributions of this study are threefold. First, this is one of the first studies to propose an AF prediction method for critically ill sepsis patients. For this purpose, we use the CinC data for training and only the MIMIC III ICU data for testing; the previous methods used only the CinC data for both training and testing. Second, we not only predict AF immediately before its onset, but also analyze how much in advance we can predict the AF by using the prior 5 min of ECG data, thus allowing adequate time for potential clinical interventions prior to AF onset in real-world scenarios. Third, we provide valuable annotations for the normal sinus rhythm (NSR)-to-AF transition subjects (pre-AF recordings) collected from the MIMIC III ICU data, which will benefit other researchers and advance AF prediction research.

## 2. Description of the Database

Two different data sets were used in this study:

### 2.1. Mimic III Database (Used Only as Testing Data)

In this study, a subset of the Medical Information Mart for Intensive Care (MIMIC) III data set was used. MIMIC III is a large open source medical record database publicly available in PhysioNet [18] which contains deidentified health-related data from patients who stayed in critical care units of the Beth Israel Deaconess Medical Center between 2001 and 2012 [19]. It includes a variety of information such as patient demographics, laboratory test results, vital sign measurements, medications, nurse and physician notes, imaging reports, and out-of-hospital mortality, which are some of the notable parameters among many others that are available. In many patients, MIMIC III links continuous ECG waveforms to a wealth of time-varying clinical and hemodynamic data. The sampling frequency of the ECG recordings was 125 Hz and the measurement unit was millivolts (mV).

We have used a total of 50 critically ill ICU patients from the MIMIC III database. Twenty-five of these subjects had non-AF to AF transition, who are henceforth referred as “AF transition subjects.” Additionally, these AF transition subjects had at least 1 h of non-AF rhythms before the AF onset. It is to be noted that the first onset of AF was adjudicated by two physicians (AW and DDM). The physicians at the University of Massachusetts Medical School and Boston University’s Medical School were involved in finding AF transition subjects.

Finding AF subjects with the above-described requirements was a manually demanding task since it required searching through thousands of patients’ ECG data records. Consequently, due to the above requirement, we found a limited number of patients for examining our algorithm’s predictive capability. From the “MIMIC III waveform database matched subset” [20], 18 subjects were identified with non-AF to AF transition. ECG signals were annotated by board-certified physicians specializing in AF management (AW and DDM). These AF transition subjects had sepsis according to the International Classification of Diseases, Ninth Revision (ICD-9) codes.

Moreover, the physicians identified seven additional subjects with non-AF to AF transition who were not included in the MIMIC III matched subset, rather only from the MIMIC III database. These seven subjects were from the critically ill group; however, since these seven subjects were not from the MIMIC III matched subset, no clinical information about sepsis was available. Overall, a total of 25 (=18 + 7) subjects with non-AF to AF transition (i.e., pre-AF) were identified.

Similarly, in order to form the control group, 25 NSR subjects were chosen to match the number of non-AF to AF transition subjects. These control subjects were randomly chosen using the previous AF and NSR annotations provided by our group, and were adjudicated to not be in AF for the entire duration of the waveform recording [21]. These 25 NSR control subjects were from a critically ill group with sepsis. As a result, the total number of subjects in this study was 50, and they were used only as the test data set. The annotated data will be made publicly available at https://biosignal.uconn.edu/resources/ to facilitate further research.

### 2.2. AFPDB Database (Used Only as Training Data)

The AFPDB data set is a publicly available paroxysmal atrial fibrillation prediction database, which originated from the PAF prediction challenge administered by Computers in Cardiology in 2001 [18,22]. The training database contains 25 pairs of ECG recordings obtained from patients with paroxysmal AF where each pair is recorded from different PAF patients. Each pair of data contains one 30-min ECG segment that ends just prior to the onset of a PAF event and another 30-min ECG segment at least 45 min distant from the onset of PAF. Moreover, recordings from 25 normal subjects were provided; each recording is 30 min long and has two channels.

For this study, we used 25 control ECGs and 25 ECG segments which are just prior to the onset of PAF (referred to as pre-AF). As a result, the 50 recordings are from different subjects. Each ECG segment contained two-channel traces from Holter recordings with a sampling rate of 128 Hz and 12-bit resolution.

## 3. Proposed Method

**Figure 1:**
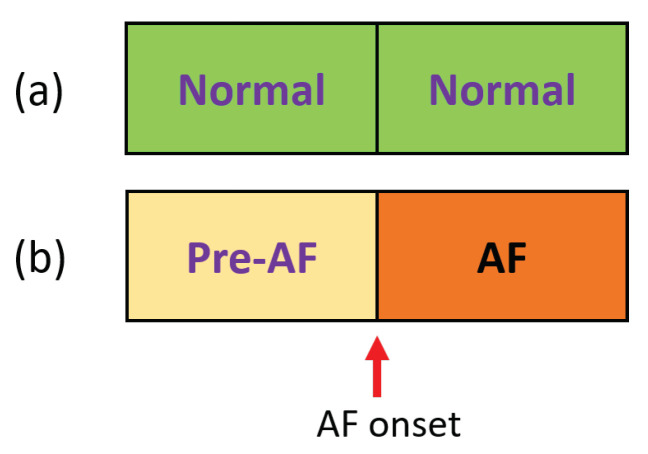
AF prediction schematic. (**a**) Normal recordings followed by normal recordings (control group). (**b**) Pre-AF (i.e., normal) recordings followed by AF onset.

The AF prediction scheme is illustrated in Figure 1. AF onset refers to the time point when AF started and the ECG recording prior to this onset is referred to as “pre-AF.” The goal of our proposed method is to be able to predict the AF onset using this “pre-AF” data. For the control group, since there is no AF event, a random ECG portion is identified as the control. The aim is to discriminate these pre-AF segments from the NSR or normal segments.

Our method consisted of first preprocessing the ECG recordings, followed by feature extraction using several standard heart rate variability (HRV) analysis methods as well as time–frequency-based analysis of the heart rate signal. Finally, the pre-AF segments/ECG data are identified from the control group using the extracted features and machine learning classifiers.

### 3.1. Preprocessing

The first step of the preprocessing is the extraction of the heart rate data from ECG recordings. For short-term heart rate analysis, a 5-min ECG segment is recommended [14,23]. For the non-AF to AF transition subjects, a 5-min segment was taken from the ECG recordings immediately prior to the AF onset. Next, the R-peaks of the ECG segment were determined by a newly developed R-peak detection method which can reconstruct the ECG from the time-frequency-based sub-band decomposition [24]. After the R–R interval series was obtained, several preprocessing steps were performed depending on the feature extraction approaches. For calculating the frequency domain heart rate features, ectopic beats were first removed using the impulse rejection method described in [25] to obtain the corrected heart rates. Next, the corrected heart rate was resampled at 4 Hz by cubic spline, which was followed by trend removal.

For the time–frequency-domain-based analysis methods (variable frequency complex demodulation (VFCDM) and tunable Q-factor wavelet transform (TQWT)), the original R–R interval series was resampled at 4 Hz by cubic spline to make the samples evenly spaced; ectopic beat removal was not performed. For the time domain and nonlinear feature extraction methods, the original R–R interval was used without any further preprocessing.

### 3.2. Feature Extraction from RR Intervals

**Figure 2:**
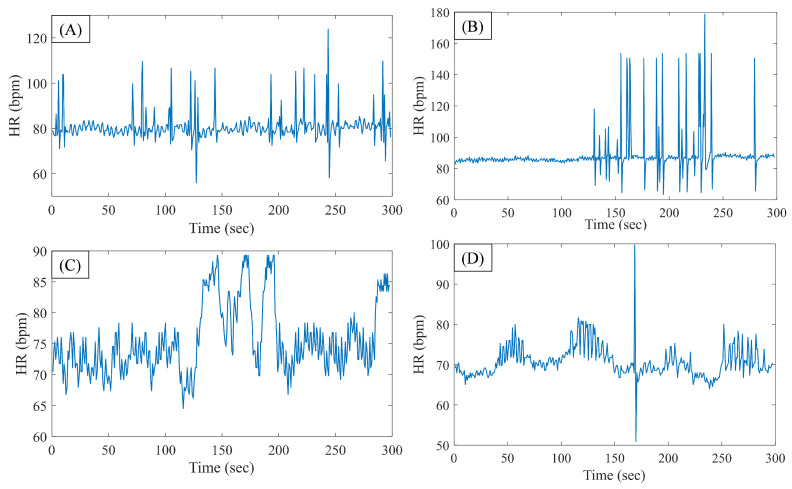
Representative 5-min heart rate signal, which is immediately prior to the AF onset: (**A**) from the CinC data set and (**B**) from the MIMIC III data set. Representative 5-min heart rate signal for control group: (**C**) from the CinC data set and (**D**) from the MIMIC III data set.

Figure 2A shows a representative 5-min heart rate signal, which is immediately prior to AF onset (from the CinC data set), whereas Figure 2B shows the same for a sample segment from the MIMIC data. Figure 2C,D show sample HRV segments from the CinC and MIMIC data, respectively, for the control group. From the pre-AF segments, it can be seen that there are several occurrences of PAC beats.

In order to predict AF, the following HR signal-based feature extraction methods were used in this study:

#### 3.2.1. Time Domain Features

From the 5-min original heart rate signal, several standard time domain HRV features were calculated. The features include: standard deviation of the heart rate data (SDNN); total number of consecutive heart rate data differences greater than 50 ms (NN50); sum of NN50 divided by the total number of RR intervals (pNN50); skewness and kurtosis of the heart rate data; and root mean square of successive differences (RMSSD) of heart rate, which is divided by the mean heart rate of the corresponding segment to counter the variability among different subjects and segments. Finally, triangular index was also calculated as a geometric HRV feature, defined as the total number of RR intervals divided by the number of RR intervals that fall into a modal bin [11,23].

#### 3.2.2. Nonlinear Features

In order to calculate the nonlinear features, the original heart rate signal was used. The extracted nonlinear HRV features include:

##### Poincaré Features

The Poincaré plot is a geometrical method that can be used to assess the dynamics of HRV. For HRV analysis, it is generated by plotting every RR interval against the prior interval, which creates a scatter plot [26]. For the Poincaré plot feature, an ellipse is fitted to the scattered points and the two following parameters are calculated for the quantification of the geometry.

$SD_{1}$ is the standard deviation of the projection of the Poincaré plot on the line perpendicular to the line of identity, which reflects the level of short-term variability. $SD_{2}$ is the standard deviation of the projection of the Poincaré plot on the line of identity, which is thought to indicate the level of long-term variability [27]. They are defined as follows, where SD is the standard deviation and $RR_{i}$ is the *i*th RR interval [28]:

(1) $$\begin{array}{c} SD_{1}=\frac{1}{\sqrt{2}}SD\left(RR_{i+1}-RR_{i}\right)\\ SD_{2}=\sqrt{2\times SD{\left(RR_{i}\right)}^{2}-0.5\times SD{\left(RR_{i+1}-RR_{i}\right)}^{2}} \end{array}$$

Moreover, the $SD_{1}/SD_{2}$ ratio was used as another Poincaré plot feature.

##### Sample Entropy

Sample entropy (SampEn) measures the randomness of the HRV signal. SampEn is defined as the negative logarithm of the conditional probability that two sequences similar for *m* points remain similar at the next point, where self-matches are excluded [29,30]. SampEn has two main parameters: template length ‘*m*’ and tolerance ‘*r*’. A lower value of SampEn indicates more self-similarity in the heart rate time series [30].

##### Multiscale Entropy

Multiscale entropy (MSE) analyzes the dynamic complexity of a system by quantifying its entropy over a range of temporal scales [31]. MSE is a two-step procedure: the first step consists of generating a coarse-grained time series by averaging the data points of the original HRV series while the second step consists of computing the sample entropy of each coarse-grained time series [32].

##### Approximate Entropy

Approximate entropy (ApEn) is the conditional probability of two segments of a time series of length *N* matching at a length m+1 if they match at a length *m* [33]. ApEn is a function of three parameters—*N*, *m*, and *r*—where *N* is the length of the HRV signal, *m* is the embedding dimension, and *r* is the tolerance/distance threshold, which is fixed to match segments when they are compared with each other [34].

##### Autoregressive (AR) Model

The RR interval time series can be described as the output of an AR model. By fitting an AR model, the fluctuations of the HRV series can be separated into those of the regulated component and the random component, which is the residual of the AR model [35].

(2) $$\begin{array}{c} RR\left(t\right)=\sum_{k=1}^{p}A\left(k\right)RR\left(t-k\right)+n\left(t\right) \end{array}$$

Here, A(k) is the AR model coefficient, n(t) is the model error or residual component, and *p* is the model order. The variance of this residual component n(t) is an estimate of the residual noise power ($\sigma_{AR}^{2}$), which is used as a feature in our work. For the pre-AF segments, this $\sigma_{AR}^{2}$ is expected to have high value due to the frequent occurrence of ectopic beats. In this study, the 12th order AR model was empirically selected.

#### 3.2.3. Frequency Domain Features

Frequency domain parameters can provide useful information about the sympathetic and parasympathetic nervous activity and are shown to be effective for predicting PAF onset [12,14]. In order to calculate the frequency domain HRV features, ectopic beat removal was performed using the McNames impulse removal filter [25]. This corrected HRV was then resampled at 4 Hz by cubic spline and trend removal.

**Figure 3:**
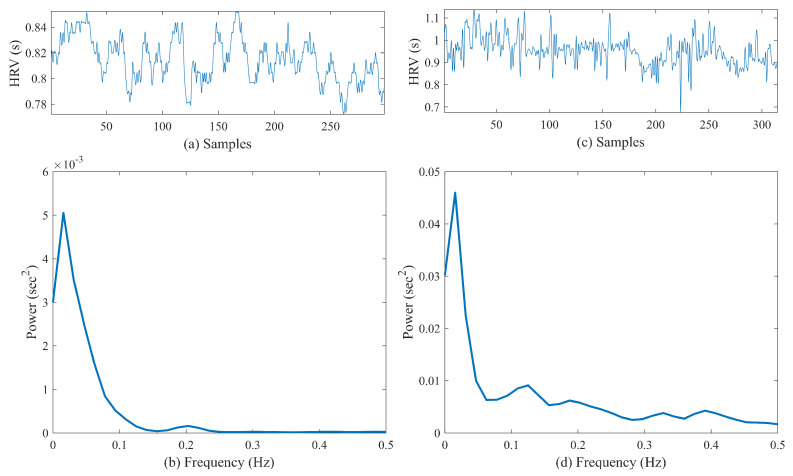
(**a**) Preprocessed heart rate signal from a control subject and (**b**) the corresponding PSD. (**c**) Preprocessed heart rate signal from a pre-AF segment and (**d**) the corresponding PSD.

The power spectra of HRV data were calculated using Welch’s periodogram method with 50% overlap. First, a Blackman window (length of 256) was applied to each segment, and then the fast Fourier transform was calculated for each windowed segment. Finally, the power spectra of the segments were averaged [36]. Figure 3a,b show a sample preprocessed heart rate signal obtained from a control subject and the corresponding PSD, respectively. Figure 3c,d show similar examples for a pre-AF segment. From the PSD, the very-low-frequency power (VLF) in the range 0–0.04 Hz, low-frequency power (LF) in the range 0–0.15 Hz, high-frequency power (HF) in the range 0.15–0.40 Hz, and total power were computed first [23]. Next, LF/HF, normalized LF (LFn = LF/total power), and normalized HF (HFn = HF/total power) were calculated and analyzed for pre-AF vs. NSR discrimination.

#### 3.2.4. VFCDM-Based Features

Variable frequency complex demodulation (VFCDM) is a high-resolution time–frequency domain method, which is widely used for various biosignal processing, including ECG [24,37], EDA [36], PPG [38,39] and other signals. First, the heart rate signal was resampled at 4 Hz to make the samples evenly spaced, which was followed by high-pass filtering (0.01 Hz) to remove any trends.

Using the VFCDM, the preprocessed heart rate signal was decomposed into *K* number of components or sub-bands [37]:

(3) $$\begin{array}{c} hrv\left(t\right)=\sum_{i=1}^{K}V_{i}\left(t\right) \end{array}$$

where hrv is the input heart rate signal, $V_{i}\left(t\right)$ is the *i*th component or sub-band, and *K* is the number of sub-bands. In this study, by applying the VFCDM, the input hrv(t) was divided into K=12 sub-bands. These sub-bands were evenly spaced in the frequency range and their frequencies depend on the sampling rate. Since the heart rate data were resampled at 4 Hz, the spectral components (i.e., $V_{i}\left(t\right)$) were centered at 0.08, 0.24, 0.40, 0.56, 0.72, 0.88, 1.04, 1.20, 1.36, 1.52, 1.68, and 1.84 Hz.

**Figure 4:**
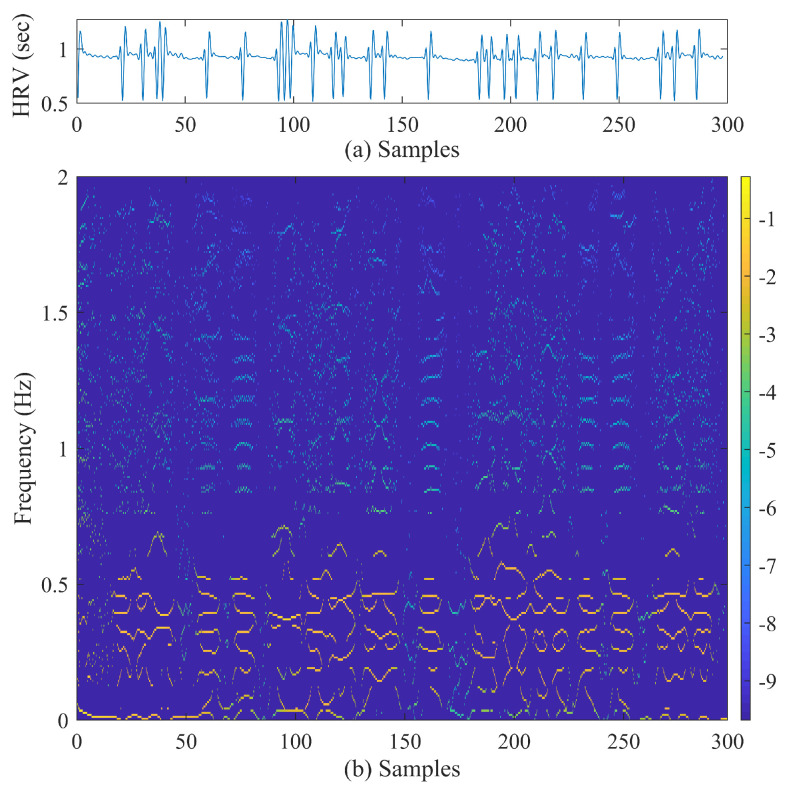
(**a**) Sample 5-min heart rate signal from a pre-AF ECG segment. (**b**) Time–frequency representation obtained using VFCDM.

Figure 4 shows a sample of preprocessed heart rate signal (the input) and the time–frequency representation obtained using the VFCDM.

From the 12 VFCDM components (3), only the third and fourth components were added to make a reconstructed heart rate time series, $hrv_{rec}\left(t\right)=V_{3}\left(t\right)+V_{4}\left(t\right))$.

**Figure 5:**
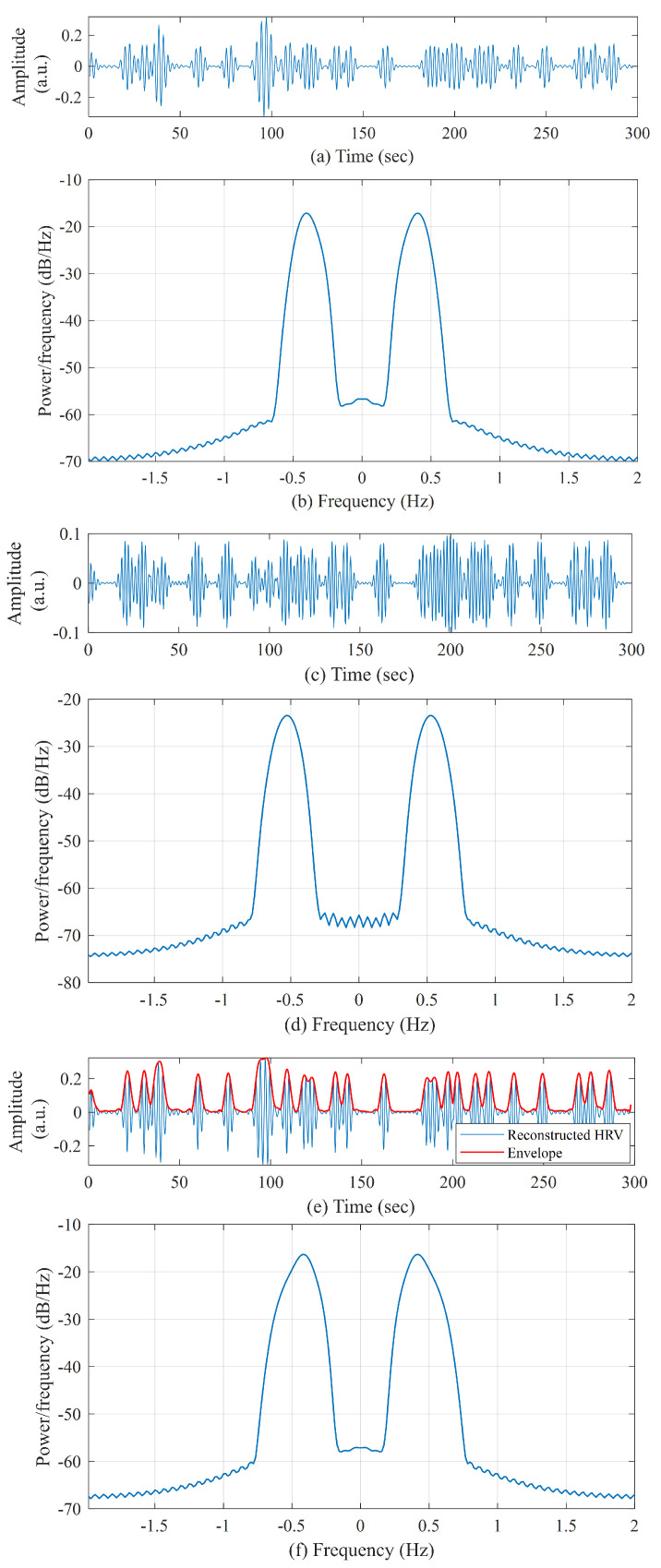
For a sample 5-min pre-AF heart rate signal: (**a**) third component of the VFCDM decomposition; (**b**) PSD of the third component; (**c**) fourth component of the VFCDM decomposition; (**d**) PSD of the fourth component; (**e**) reconstructed heart rate signal along with the Hilbert transform envelope; and (**f**) PSD of the reconstructed heart rate.

This reconstructed heart rate time series ($hrv_{rec}$) contained the high-frequency components and was found to be highly useful for analyzing the heart rate variation due to frequent ectopic beats and subsequently, for AF prediction when compared to the control group. Figure 5a–d shows the third and fourth components obtained from the VFCDM decomposition of a sample heart rate signal (pre-AF group) and their respective power spectral density (PSD). The PSDs shows that third component is centered at 0.40 Hz, whereas the fourth component is centered at 0.56 Hz, which surrounds the HF part of HRV and represents the variation due to ectopic beats. Figure 5e shows the reconstructed heart rate signal ($hrv_{rec}$) and Figure 5f shows the PSD of the reconstructed HRV.

Using this reconstructed heart rate signal, we performed the Hilbert transform to obtain the signal envelope as follows [36]:

(4) $$\begin{array}{c} H\left(t\right)=\frac{1}{\pi }P\int_{-\infty }^{\infty }\frac{hrv_{rec}\left(\tau \right)}{t-\tau }d\tau \end{array}$$

where *P* indicates the Cauchy principal value. $hrv_{rec}\left(t\right)$ and H(t) form the complex conjugate pair, which can be used to define the analytic signal A(t):

(5) $$\begin{array}{c} A\left(t\right)=hrv_{rec}\left(t\right)+iH\left(t\right)=a\left(t\right)e^{j\theta (t)} \end{array}$$

where

(6) $$\begin{array}{c} a\left(t\right)={\left[hrv_{rec}^{2}\left(t\right)+H^{2}\left(t\right)\right]}^{1/2}\\ \theta \left(t\right)=\arctan\;[H\left(t\right)/hrv_{rec}\left(t\right)] \end{array}$$

The a(t) is considered the instantaneous amplitude or envelope of A(t). Figure 5e shows the reconstructed HRV ($hrv_{rec}$) and the Hilbert transform envelope or instantaneous amplitude (a(t)). From this instantaneous amplitude, mean, variance, and energy were calculated as features.

#### 3.2.5. TQWT-Based Features

The tunable Q-factor wavelet transform (TQWT) is a flexible full-discrete wavelet transform, which is suitable for analyzing oscillatory signals [40]. TQWT facilitates analysis of oscillatory signals using three adjustable parameters: Q-factor (*Q*), redundancy or total oversampling rate (*r*), and the number of decomposition levels (*J*). *Q* controls the number of oscillations of the wavelet and affects the extent to which the oscillations of the wavelet are sustained [41]. *r* helps to localize the wavelet in the time domain without affecting its shape.

For a certain decomposition level *J*, TQWT decomposes an input signal into J+1 sub-bands. It is performed by iteratively applying the two-channel filter bank on its low-pass channel. TQWT consists of a sequence of two-channel filter banks, with the low-pass output of each filter bank being used as the input to the successive filter bank [42].

For a low oscillatory signal, *Q* will be lower, whereas a higher *Q* value is required for high oscillatory signals. As a result, the wavelets will be more oscillatory with narrower frequency response. Unwanted excessive ringing of wavelets needs to be prevented while performing TQWT by appropriately choosing the value of *r*, which is recommended to be greater than or equal to 3. Details about TQWT can be found in [40,41]. In order to extract AF predicting features from the HRV signal using TQWT, J=17, Q=3, and r=4 have been selected empirically in this study.

**Figure 6:**
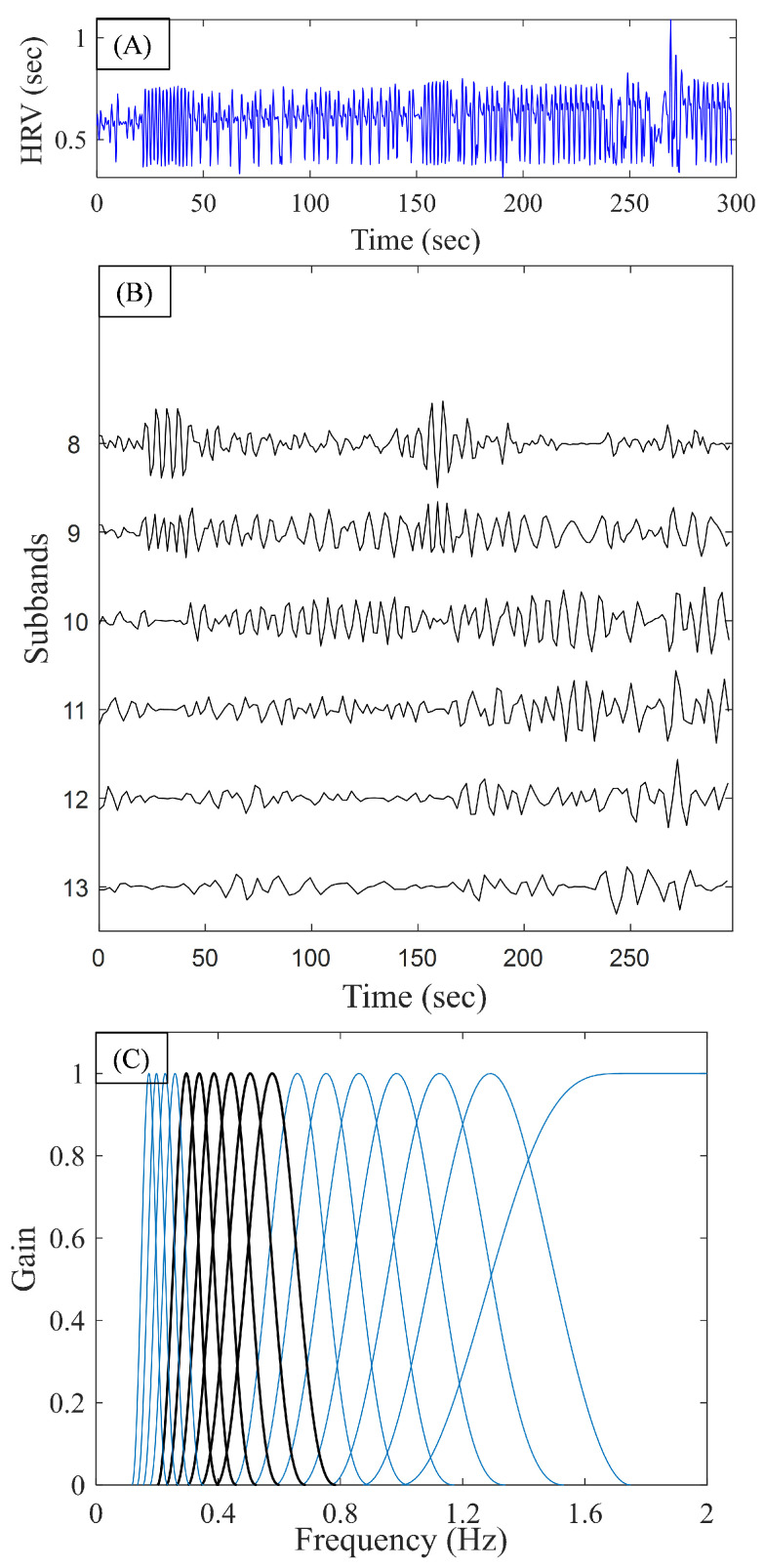
(**A**) Sample 5-min heart rate signal. (**B**) TQWT coefficients obtained from levels 8 to 13. (**C**) Frequency response of the TQWT transform with the selected parameters (normalized to have unity gain).

Figure 6A,B shows the input heart rate signal (from a pre-AF segment) and the TQWT coefficients, respectively, obtained from levels J=8 to J=13 where the resampled heart rate signal was used as the input. Figure 6C shows the frequency response of the TQWT transform for the selected parameters where the gain is normalized to have unity amplitude. Since J=17 was used, the center frequencies of the TQWT sub-bands were (in descending order): 2 Hz, 1.31 Hz, 1.15, 1 Hz, 0.88 Hz, 0.77 Hz, 0.67 Hz, 0.59 Hz, 0.52 Hz, 0.45 Hz, 0.39 Hz, 0.35 Hz, 0.30 Hz, 0.26 Hz, 0.23 Hz, 0.20 Hz, and 0.18 Hz. The frequencies corresponding to *J* = 8 to *J* = 13 are marked in black in Figure 6C.

We analyzed the mean, variance, energy, entropy, and spectral entropy calculated from the coefficients of different sub-bands and found that energy as well as spectral entropy were the most useful ones as the discriminating features to be used for pre-AF and control segments.

Spectral entropy is a generalization of information entropy and it measures the distribution of frequencies. Spectral entropy treats the signal’s normalized power distribution in the frequency domain as a probability distribution and calculates Shannon entropy from it [43,44]. For a given time–frequency spectrogram S(t,f), the probability distribution at time *t* is given by:

(7) $$\begin{array}{c} P\left(t,m\right)=\frac{S(t,m)}{\sum_{f}S\left(t,f\right)} \end{array}$$

The instantaneous spectral entropy at time *t* is calculated as [44]:

(8) $$\begin{array}{c} H\left(t\right)=-\sum_{k=1}^{N}P\left(t,k\right)\log_{2}P\left(t,k\right) \end{array}$$

In order to obtain a scalar feature value, $L_{2}$ norm of this instantaneous spectral entropy was used as the feature (referred to as “ENT”).

### 3.3. AF Prediction Framework

After several features were extracted from the five different domains, suitable features were selected by visual analysis (scatter plots and box plots) as well as cross-validation on the training data.

Based on the analysis performed using the training data, 14 features were selected. The selected features for the machine learning model include RMSSD; $SD_{1}$; AR residual noise; variance from VFCDM; LF/HF; LFn; TQWT spectral entropy from bands 8, 11, 12, and 13; and TQWT energy from sub-bands 9, 10, 11, and 12.

Several machine learning classifiers were analyzed using the selected feature subset and the performance of those classifiers is described in the Results section. Finally, support vector machine (SVM) and random forest (RF) were chosen for our AF prediction. SVM is a popular and well-established method for binary classification problems where a maximum margin between the training and test data is constructed [45]. RF classifier is formed by combining multiple randomly constructed tree models [46]. In the bagging (bootstrap aggregation) learning concept, many weak learners are trained over subsets drawn with replacements from the training set and their outputs are voted to determine a predictive estimate. This is shown to decrease the variance of the model without increasing the bias, thus resulting in diverse ensembles [47].

**Figure 7:**
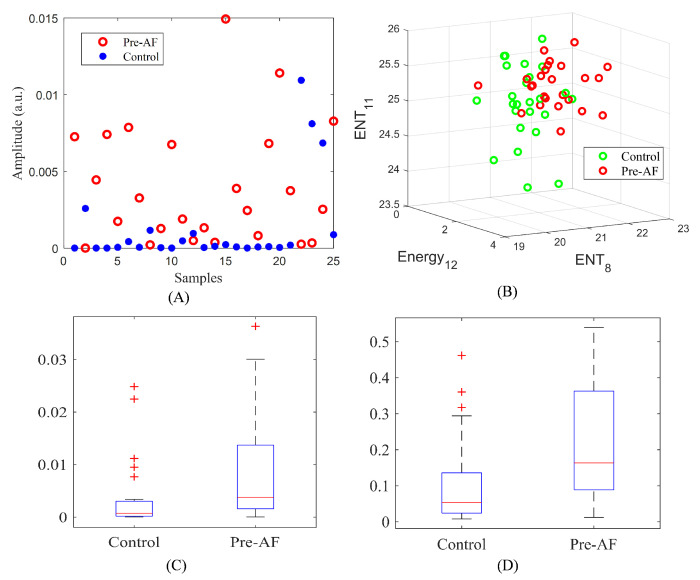
(**A**) VFCDM feature. (**B**) 3D scatter plot of spectral entropy (level 8, 11) and energy of level 12. (**C**) Box plots of RMSSD. (**D**) Box plots of AR residual noise.

**Figure 8:**
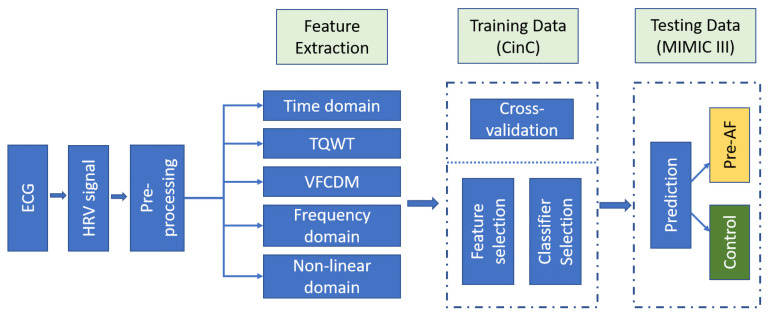
Overview of the proposed AF prediction method.

Figure 7A shows the scatter plot for the variance of VFCDM, whereas Figure 7B shows the 3D scatter plot for TQWT energy and spectral entropy. The scatter plots show that control and pre-AF samples have some visible separation for most of the cases. Figure 7C,D show the box plots for RMSSD and AR residual noise. The box plots have nonoverlapping medians, indicating the discriminatory property of the features. Figure 8 shows the complete flowchart of the proposed AF prediction method.

## 4. Results

In order to evaluate the prediction performance of our proposed method, commonly used binary classification accuracy measures were used. An ECG segment prior to the AF onset was denoted as a positive class, whereas an ECG segment from the control group was referred to as a negative class.

(9) $$\begin{array}{cc} \; & Sensitivity\;(SEN)=TP/(TP+FN)\\ \; & Specificity\;(SPE)=TN/(TN+FP)\\ \; & Accuracy\;(ACC)=(TP+TN)/(TP+FN+TN+FP)\\ \; & Positive\;\;predictive\;\;value\;(PPV)=TP/(TP+FP)\\ \; & Negative\;\;predictive\;\;value\;(NPV)=TN/(TN+FN) \end{array}$$

where TP denotes the number of true positives, TN is the number of true negatives, FP is a false positive, and FN is a false negative.

### 4.1. Results on Training Data (CinC Data)

From the CinC data set, we have 25 control and 25 pre-AF ECG segments. With these 50 segments, the well-established *k*-fold cross-validation was performed to select the classifier model and tune the hyperparameters. The training data were split into *K* disjoint partitions (K=5) and each time (K−1) folds were used for training while the last fold was treated as test data; the entire process was repeated *k* times [44]. For this study, we have explored several machine learning classifiers including support vector machine (SVM), discriminant analysis (DA), *k*-nearest neighbor (kNN) and random forest (RF). For the discriminant analysis, both linear and quadratic boundaries along with Mahalanobis distance were analyzed. Moreover, diagonal linear and diagonal quadratic discriminant functions were also used (referred to as “diaglinear” and “diagquadratic”), which are similar to linear and quadratic discriminant functions except the estimate of the covariance matrix is diagonal [48]. For SVM, both the linear and radial basis function (RBF) kernels were used. In *k*NN, both Euclidean and Cityblock (Manhattan) distance were used with the variation of “*K*” values, which denotes the number of the nearest neighbors to be used. For the RF, the hyperparameters were varied during the fivefold cross-validation and it was found that with the selected feature subset, 50 trees resulted in the best prediction performance. Table 1 shows the performance of several machine learning classifiers using the training data (CinC) for the fivefold cross-validation.

It can be seen from Table 1 that the SVM and RF models resulted in better performance than the rest. The confusion matrices for both SVM and RF are shown in Table 2. With the fivefold cross-validation, the RF classifier achieved 80% sensitivity, 76% specificity, and 78% accuracy on the training data, whereas the SVM obtained 76% accuracy, sensitivity, and specificity.

### 4.2. Results on Test Data (MIMIC III ICU)

Next, the trained model was tested using the critically ill ICU data from MIMIC III. It is to be noted that the feature subset and model parameters were fixed by doing the cross-validation on the training data (CinC); the trained model was blindly tested on the ICU data. The test data set contained 25 ECG recordings from the subjects with no-AF to AF transition (pre-AF segments) and 25 control ECG recordings.

#### 4.2.1. Test Results on the Data Prior to AF Onset

First, the model was tested using the ECG data, which are immediately prior to the AF onset. These immediately prior data are expected to exhibit the most AF-predicting properties. Table 3 shows the confusion matrices for these test data using both the RF and SVM classifiers. The RF classifier identified 20 pre-AF segments correctly, resulting in sensitivity of 80%. Moreover, for the control group, RF detected 24 segments correctly, resulting in 96% specificity and an overall 88% accuracy. The radial basis SVM achieved 80% sensitivity, 100% specificity, and 90% accuracy.

In order to demonstrate the efficacy of our proposed method, we compared its performance with the Narin et al. method [14]. Narin et al. reported two different models: one is for PAF as well as the control subjects (model 1), whereas the other is only for the PAF subjects (model 2). Model 1 consisted of RMSSD, NN20, pNN20, $FFT_{VLF}$, and $FFT_{HF}$ features; the *k*NN classifier is reported in Narin et al. [14]. The second model used RMSSD, $FFT_{VLF}$, $FFT_{LF}$, and total power of FFT along with the *k*NN classifier [14]. In order to compare the performance, both of these models were trained and tested using the same data that we used (CinC and MIMIC, respectively) and the resulting confusion matrices are presented in Table 4. From the table, it can be seen that both of the reported models of [14] have low sensitivity compared to ours (Table 3).

#### 4.2.2. Test Results for Moving Backward from AF Onset

**Figure 9:**
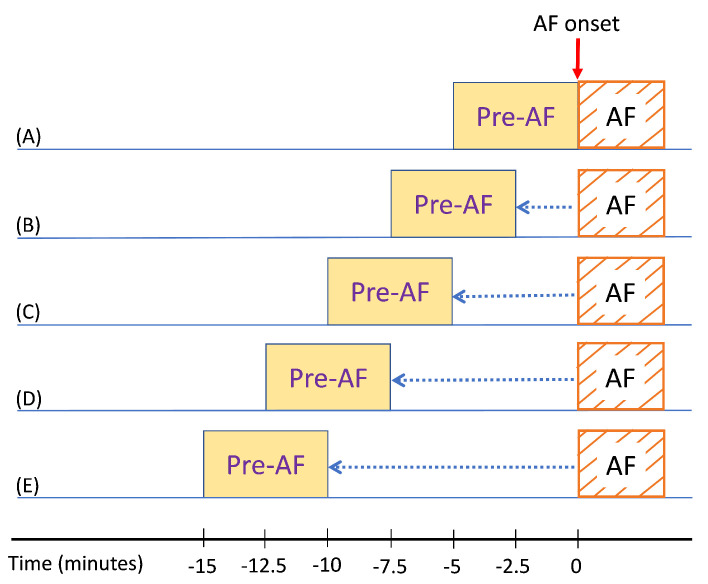
Illustration of AF prediction for moving backward in time from the onset.

In the next step, we analyzed how much in advance in time we can predict the AF onset. We analyzed how the prediction performs if we started far before AF onset. In order to study this, we took 5-min ECG segments and moved backward in a 50% overlap all the way up to 15 min prior to AF onset. As a result, the algorithm was tested using the ECG data from 2.5 min, 5 min, 7.5 min, and 10 min prior to AF onset. Figure 9 illustrates this testing scenario; for example, Figure 9E shows that one prediction was performed using the ECG data that was from 15 to 10 min prior to the AF onset.

For each of the testing scenarios demonstrated in Figure 9, we tested the already trained classifiers as mentioned in the previous subsection. Table 5, Table 6, Table 7 and Table 8 show the confusion matrices from testing the critically ill ICU ECG data for the four different scenarios illustrated in Figure 9B–E. For each scenario, the results are presented using both RF and SVM classifiers.

It can be seen from Table 5 that when 2.5 min prior to AF onset ECG data were used for AF prediction, the RF model predicted 18 pre-AF segments correctly; the prediction was correct for 24 segments for the control class, resulting in 84% accuracy. However, the prediction performance slightly degraded as we moved farther from the AF onset. When we used the ECG data from 10 min before the AF onset, the AF prediction sensitivity, specificity, and accuracy were 72%, 88%, and 80%, respectively.

Finally, in Table 9 the sensitivity, specificity, accuracy, PPV, and NPV for different time durations are reported for both of SVM and RF classifiers. Although for the immediately prior to AF data SVM had slightly higher accuracy than did RF, for all other durations RF had better performance than did the SVM. Moreover, we compared the performance of our presented method with Narin et al. [14] for different time durations. For the comparison, we extracted the reported features described in [14], trained the *k*NN classifier on the CinC data, and tested the model using the MIMIC III ICU data. From the table, it is evident that our proposed method achieved better performance than the compared method for all cases.

## 5. Discussion

We presented a novel approach to predict AF during sepsis from critically ill ICU patients using the RR interval variability of ECG. Since the frequent occurrence of premature ectopic beats is shown to be a predictor of AF, the HRV-derived features were well suited for describing the variability.

In order to use this variability due to frequent occurrence of ectopic beats, ectopic beat removal was not used for preprocessing the HRV signal for the time domain, time–frequency domain, or nonlinear methods. Ectopic removal was used only for the frequency domain feature extraction, as this is the standard procedure for calculating frequency domain HRV features [17,23].

In this work, we extracted several features from 5-min heart rate signals using five different methods: time domain, VFCDM, TQWT, nonlinear, and frequency domain. With the extracted features, we trained machine learning models using the CinC data and performed cross-validation to select suitable features as well as the model parameters. Once we obtained the highest accuracy using the training data, we directly applied the trained model to the ICU data. For different combinations of the extracted features, we performed cross-validation using the training data and measured the prediction accuracy. Next, we chose the combination of classifier model and associated features which provided the best training performance. When other features were selected, the prediction accuracy on the training data was lower. Finally, our proposed method achieved reasonable performance on this blind test data, which shows the efficacy of our method.

For the performance comparison, the Narin et al. [14] method was chosen for a few reasons. First, unlike most other methods, the authors of [14] used normal subjects along with the PAF subjects. They performed the cross-validation performance using both the normal and PAF subjects, and not only the PAF subjects. Second, they analyzed how early they could predict AF by going backward in time. Finally, their method studied 5-min HRV signals to predict PAF. However, similar to most other reported AF prediction methods, no evaluation using an external test data set was performed. The fact that our method achieved higher performance than [14] for all the different time durations clearly shows the efficacy of the presented method. Moreover, this reflects that overfitting can be an issue when only the cross-validation results are reported using a small data set without doing an external test set.

There are three main contributions of our study: we tested AF prediction using a new and different data set, which consists of critically ill sepsis patients. After obtaining good prediction accuracy for the ECG data immediately prior to the AF onset, we analyzed how much in advance we could predict the AF. We achieved 80% overall accuracy for predicting AF 10 min prior to its onset. Currently, we do not have interventions to effectively prevent AF. Hence, the ability to predict AF will enable enrichment of trials of interventions to prevent AF. While 10 min of notice ahead of AF occurrence would be tight to institute an AF preventive strategy in practice, it may be enough time to give an intervention in an experimental setting. This work is foundational for predicting AF with longer duration. Most importantly, our work would help minimize the amount of time a patient spends in AF, as reducing the time burden of a patient’s AF to only a few minutes may mitigate their risk for ischemic stroke.

Additionally, though the accuracy expectedly trends downward further away from AF onset, this decrease is largely a function of the algorithm’s sensitivity, and the specificity actually remains high in all time windows examined. Therefore, this suggests that our approach can be especially useful for confirming true positive cases given a positive result. Finally, we provided new annotations for other researchers, which can be used as a valuable resource for future work in AF prediction.

Our study is different than the CinC 2001 data-based works [7,8,9,10,11,12,13,14,15,16,17]. In CinC-based works, AF prediction was analyzed using only the PAF subjects. In other words, within the PAF subjects, the AF prediction analysis was performed where each subject had two recordings: pre-AF and distant from AF. However, in this study, we performed AF prediction using the control subjects and the critically ill ICU sepsis subjects who had a transition from non-AF to AF. As a result, the pre-AF and control segments are from different subjects, which is distinct from the CinC data set.

Finally, our findings should be considered in light of study limitations. The main limitation is that we had a relatively small sample size from the MIMIC III ICU data. Moreover, for the few MIMIC III transition subjects (which were not in the matched subset), we were unable to determine the sepsis status due to the lack of available clinical information. However, given the scarcity of the AF prediction data, it is understandable that getting this kind of rare data can be difficult. As a result, we provide our data annotations for other researchers so that people can use this data for advancing AF prediction research. Our work can be viewed as a preliminary study wherein we showed that by using the RR interval variation characteristics, we can achieve satisfactory AF prediction accuracy for critically ill ICU patients. Future works can focus on validation using a larger database when it becomes available and analyze whether the AF prediction performance differs between sepsis and nonsepsis ICU patients. Moreover, we aim to extend the prediction timeframe to further in advance in order to give an even more comfortable margin for taking action. Although our algorithm was validated on ECG data collected in the ICU with the standard ECG electrodes and leads, it is equally effective for any ECG modality including patch electrodes and wearables, including Holter, loop recorder, and implantable devices. Our algorithm uses the variability and morphology of the ECG to predict AF, hence, any ECG modality will suffice.

## 6. Conclusions

In this study, we have presented a novel approach to predict AF from critically ill sepsis patients using the MIMIC III ECG data. We have extracted various features from 5-min heart rate signals using time domain, frequency domain, nonlinear, VFCDM, and TQWT methods. With a subset of selected features, we have trained RF and SVM models using the CinC data; next, the trained models were directly applied to the MIMIC III ICU data without any further tuning. The proposed algorithm achieved good AF prediction performance on the test data and when compared with a state-of-the-art method, our method achieved better accuracy, thus showing the effectiveness of the presented method for real-life ICU data. Moreover, we analyzed how much in advance we can predict AF using the heart rate data. Since this is the first work to predict new onset AF in critically ill sepsis patients, we provide our annotations of the MIMIC III data to facilitate further AF prediction research. Future studies can explore how the AF prediction differs between sepsis and nonsepsis patients as well as validating the method using a larger number of AF subjects.

## Figures and Tables

**Table 1:** Performance comparison of different classifiers.

Classifier	Type/	Sensitivity	Specificity	Accuracy
Name	Hyperparameter	(%)	(%)	(%)
DA	Linear	64	72	68
	Diaglinear	60	76	68
	Quadratic	88	60	74
	Diagquadratic	52	80	66
	Mahalanobis	92	52	72
SVM	Linear	64	72	68
	RBF	**76**	**76**	**76**
KNN	*K* = 5,	60	60	60
	Euclidean			
	*K* = 5,	64	60	62
	Cityblock			
RF	50 trees	**80**	**76**	**78**

**Table 2:** Confusion matrix on the training data.

SVM				RF			
		Predicted Label				Predicted Label	
		Pre-AF	Control			Pre-AF	Control
True Label	Pre-AF	19	6	True Label	Pre-AF	20	5
	Control	6	19		Control	6	19

**Table 3:** Confusion matrix on the test data (immediately before onset).

SVM				RF			
		Predicted Label				Predicted Label	
		Pre-AF	Control			Pre-AF	Control
True Label	Pre-AF	20	5	True Label	Pre-AF	20	5
	Control	0	25		Control	1	24

**Table 4:** Test results of the compared methods.

Model 1 [14]				Model 2 [14]			
		Predicted Label				Predicted Label	
		Pre-AF	Control			Pre-AF	Control
True Label	Pre-AF	15	10	True Label	Pre-AF	16	9
	Control	4	21		Control	3	22

**Table 5:** Confusion matrix on the test data (2.5 min before onset).

SVM				RF			
		Predicted Label				Predicted Label	
		Pre-AF	Control			Pre-AF	Control
True Label	Pre-AF	16	9	True Label	Pre-AF	18	7
	Control	0	25		Control	1	24

**Table 6:** Confusion matrix on the test data (5 min before onset).

SVM				RF			
		Predicted Label				Predicted Label	
		Pre-AF	Control			Pre-AF	Control
True Label	Pre-AF	15	10	True Label	Pre-AF	18	7
	Control	1	24		Control	3	22

**Table 7:** Confusion matrix on the test data (7.5 min before onset).

SVM				RF			
		Predicted Label				Predicted Label	
		Pre-AF	Control			Pre-AF	Control
True Label	Pre-AF	13	12	True Label	Pre-AF	18	7
	Control	2	23		Control	4	21

**Table 8:** Confusion matrix on the test data (10 min before onset).

SVM				RF			
		Predicted Label				Predicted Label	
		Pre-AF	Control			Pre-AF	Control
True Label	Pre-AF	16	9	True Label	Pre-AF	18	7
	Control	2	23		Control	3	22

**Table 9:** Confusion matrix on the test data (7.5 min before onset).

Prior	Method	SEN	SPE	ACC	PPV	NPV
Duration		(%)	(%)	(%)	(%)	(%)
0 min	SVM	80	100	**90**	100	83.33
	RF	80	96	88	95.24	82.76
	Method in [14]	60	84	72	78.95	67.74
2.5 min	SVM	64	100	82	100	73.53
	RF	72	96	**84**	94.74	77.42
	Method in [14]	56	84	70	77.78	65.63
5 min	SVM	60	96	78	93.75	70.59
	RF	72	88	**80**	85.71	75.86
	Method in [14]	52	88	70	81.25	64.71
7.5 min	SVM	52	92	72	86.67	65.71
	RF	72	84	**78**	81.82	75
	Method in [14]	36	84	60	69.23	56.76
10 min	SVM	64	92	78	88.89	71.88
	RF	72	88	**80**	85.71	75.86
	Method in [14]	68	80	74	77.27	71.43

## Data Availability

The annotations of patients’ AF transition information will be made publicly available at https://biosignal.uconn.edu/resources/.
